# Correction

**DOI:** 10.1080/20008066.2025.2476823

**Published:** 2025-03-25

A journey towards a trauma informed and responsive Justice system: the perspectives and experiences of senior Justice workers

Nicola Cogan, Dwight Tse, Melanie Finlayson, Samantha Lawley, Jacqueline Black, Rhys Hewitson, Suzanne Aziz, Helen Hamer and Cherrie Short

*European Journal of Psychotraumatology*

https://doi.org/10.1080/20008066.2024.2441075

When the article was first published online, author Christiana Stergiou was omitted from the author list in error, and the References section contained errors.

These errors have been corrected.

